# Supplementary material for: Empirical methods for controlling false positives and estimating confidence in ChIP-Seq peaks
Source: BMC Bioinformatics. 2008 Dec 5;9:523. doi: 10.1186/1471-2105-9-523 (PMC2628906; doi:10.1186/1471-2105-9-523)
Supplement: Additional File 1 — A variety of html documents from the USeq web site detailing the available applications, their best usage, output file type descriptions, command line menus, etc. [file 1471-2105-9-523-S1.zip › USeqUserGuides/JavaDoc.zip!/JavaDoc/edu/utah/seq/data/class-use/SmoothingWindow.html]

Uses of Class edu.utah.seq.data.SmoothingWindow


|  |  |  |
| --- | --- | --- |
| **Overview** \| **Package** \| **Class** \| **Use** \| **Tree** \| **Deprecated** \| **Index** \| **Help** | |  |
| PREV   NEXT | **FRAMES**    **NO FRAMES**     **All Classes** |  |


---


## **Uses of Class edu.utah.seq.data.SmoothingWindow**

| Packages that use SmoothingWindow | |
| --- | --- |
| **edu.utah.seq.analysis** | Primary analysis applications and helper classes for working with binary PointData, xxx.bar.zip files. |
| **edu.utah.seq.data** | Helper classes for working with binary PointData, EnrichedRegions, etc. |

Uses of SmoothingWindow in edu.utah.seq.analysis

| Methods in edu.utah.seq.analysis that return SmoothingWindow | |
| --- | --- |
| `SmoothingWindow` | `EnrichedRegionMaker.fetchGoodWindow()` |
| `static SmoothingWindow` | `PeakPicker.findHighestPeak(SmoothingWindow[] sm, int scoreIndex)`             Find the window with the largest score. |

| Methods in edu.utah.seq.analysis with parameters of type SmoothingWindow | |
| --- | --- |
| `void` | `ScanSeqs.calculatePoissonPValues(int maxTotal, SmoothingWindow[] sm, int scoreIndexToConvert, int pValueIndexToPlace, double lambda)`             Makes a call to R to use it to calculate Bonferroni corrected Poisson pvalues. |
| `boolean` | `EnrichedRegionMaker.checkScore(SmoothingWindow win)` |
| `static SmoothingWindow` | `PeakPicker.findHighestPeak(SmoothingWindow[] sm, int scoreIndex)`             Find the window with the largest score. |
| `void` | `ScanSeqs.savePointAndHeatMapData(java.io.File saveDir, int scoreIndex, int scoreID, SmoothingWindow[] sm, Info info, boolean twoColor)`             Saves point and heat map bar files of a given score. |
| `void` | `ScanSeqs.saveSmoothedHeatMapData(int scoreIndex, SmoothingWindow[] sm, Info info, java.io.File dir, java.lang.String description, java.lang.String units, boolean twoColor)` |
| `void` | `ScanSeqs.saveSmoothedPointData(int scoreIndex, SmoothingWindow[] sm, Info info, java.io.File dir, java.lang.String description, java.lang.String units)` |

Uses of SmoothingWindow in edu.utah.seq.data

| Methods in edu.utah.seq.data that return SmoothingWindow | |
| --- | --- |
| `SmoothingWindow[]` | `HeatMapMakerPosNeg.filterWindows(SmoothingWindow[] windows)`             Tosses any windows within the min max filter range. |
| `SmoothingWindow` | `EnrichedRegion.getBestSubWindow()` |
| `SmoothingWindow` | `EnrichedRegion.getBestWindow()` |
| `SmoothingWindow[]` | `SmoothingWindowInfo.getSm()` |
| `SmoothingWindow[]` | `EnrichedRegion.getSubWindows()` |

| Methods in edu.utah.seq.data with parameters of type SmoothingWindow | |
| --- | --- |
| `boolean` | `HeatMapMaker.baseOverlapsWindow(int bp, SmoothingWindow win)`             Is a given base contained within the window? Interbase coordinates so last window position is not included. |
| `SmoothingWindow[]` | `HeatMapMakerPosNeg.filterWindows(SmoothingWindow[] windows)`             Tosses any windows within the min max filter range. |
| `PointData` | `HeatMapMakerPosNeg.makeHeatMapPositionValues(SmoothingWindow[] win)`             Makes a stair step heat map from an array of windows in bar format. |
| `PointData` | `HeatMapMaker.makeHeatMapPositionValues(SmoothingWindow[] windows, boolean oneDecimal)`             Returns a PointData object with loaded position and values representing a top score heatmap/ stairstep. |
| `boolean` | `HeatMapMakerPosNeg.overlapOrAbut(SmoothingWindow left, SmoothingWindow right)`             Assumes right window is to right of left or abuts or doesn't overlap. |
| `void` | `EnrichedRegion.setBestSubWindow(SmoothingWindow bestSubWindow)` |
| `void` | `EnrichedRegion.setBestWindow(SmoothingWindow bestWindow)` |
| `void` | `SmoothingWindowInfo.setSm(SmoothingWindow[] sm)` |
| `void` | `EnrichedRegion.setSubWindows(SmoothingWindow[] subWindows)` |

Constructors in edu.utah.seq.data with parameters of type SmoothingWindow

`EnrichedRegion(SmoothingWindow window,
Info info)`

`EnrichedRegion(SmoothingWindow window,
int halfReadLength)`

`SmoothingWindowInfo(SmoothingWindow[] sm,
Info info)`

---


|  |  |  |
| --- | --- | --- |
| **Overview** \| **Package** \| **Class** \| **Use** \| **Tree** \| **Deprecated** \| **Index** \| **Help** | |  |
| PREV   NEXT | **FRAMES**    **NO FRAMES**     **All Classes** |  |


---
